# Bladder washing cytology in the initial evaluation of patients with neuro‐urological disorders: to do or not to do?

**DOI:** 10.1111/bju.70422

**Published:** 2026-08-16

**Authors:** Fabienne Lehner, Sarah Dugas, Veronika Birkhäuser, Oliver Gross, Lorenz Leitner, Ulrich Mehnert, Lara Stächele, Collene E. Anderson, Thomas M. Kessler

**Affiliations:** ^1^ Department of Neuro‐Urology Balgrist University Hospital, University of Zürich Zürich Switzerland; ^2^ Spinal Cord Injury Center, Balgrist University Hospital University of Zürich Zürich Switzerland; ^3^ Swiss Paraplegic Research Nottwil Switzerland

**Keywords:** urinary cytology, urinary bladder neoplasms, cystoscopy, neurogenic urinary bladder, bladder cancer

## Abstract

**Objectives:**

To evaluate if bladder washing cytology (BWC) provides a diagnostic benefit in the initial evaluation of patients with neuro‐urological disorders.

**Patients and Methods:**

This prospective cross‐sectional study investigated a consecutive series of patients with lower urinary tract symptoms referred for neuro‐urological evaluation at our department of neuro‐urology who underwent urethro‐cystoscopy (UC) and BWC from 2018 to 2023.

**Results:**

Among 581 neurological patients, 274 (47.2%) were females. Tumour‐suspicious findings leading to surgical intervention were detected in 17 (2.9%) by UC alone and in three (0.5%) by BWC but unsuspicious UC, respectively. Overall, relevant histological findings after transurethral bladder surgery (TUBS) were detected in eight (1.4%, 95% confidence interval [CI] 0.6–2.7%) patients: keratinising squamous metaplasia (two) and non‐muscle‐invasive urothelial carcinoma (six), all were initially identified by UC. Three patients refused surgery, two of whom had positive BWC without suspicious lesion in UC. Sensitivity and specificity of BWC referenced to UC alone were 0% (95% CI 0.0–36.9%) and 99.7% (95% CI 98.7–99.9%), respectively. Adding BWC to UC increased costs by 22.8% without improving tumour detection. The main study limitation was the lack of true positives in BWC.

**Conclusions:**

Bladder washing cytology in addition to UC is not justified in the initial evaluation of patients with neuro‐urological disorders as decision making regarding TUBS is based on UC findings. Moreover, omission of BWC saves relevant costs without compromising patient safety.

**Abbreviations**
BCa: bladder cancer
BWC: bladder washing cytology
CIS: carcinoma *in situ*
EAU: European Association of Urology
IQR: interquartile range, Q1–Q3
KEK: Kantonale Ethikkomission
MS: multiple sclerosis
NLUTD: neurogenic lower urinary tract dysfunction
PD: Parkinson's disease
SCC: squamous cell carcinoma
SCI: spinal cord injury
TUBS: transurethral bladder surgery
UC: urethro‐cystoscopy

## Introduction

Neurogenic lower urinary tract dysfunction (NLUTD) is a common consequence of neurological disorders such as cerebrovascular accidents, multiple sclerosis (MS), Parkinson's disease (PD), spinal cord injury (SCI), spina bifida, and others and leads to impaired bladder storage and voiding function [1, 2]. Beyond the functional impairment and risk of possible upper tract deterioration, which are assessed by video‐urodynamics, patients with NLUTD are at risk of structural complications, including bladder wall changes, stone formation, and, most critically, an increased risk of bladder cancer (BCa) due to chronic inflammation, recurrent UTIs, and long‐term catheterisation [3, 4]. Therefore, a thorough initial neuro‐urological evaluation is essential in managing patients with NLUTD to prevent complications in the future and guide therapeutic interventions [5].

Urethro‐cystoscopy (UC) plays a pivotal role in the evaluation of structural abnormalities [2]. In clinical practice, the endoscopic assessment may include bladder washing cytology (BWC), a non‐invasive method used to detect exfoliated malignant urothelial cells. BWC has a high specificity, particularly for high‐grade urothelial cancer, but limited sensitivity for low‐grade tumours [6, 7]. While the role of BWC is well established in the general population with suspected urothelial carcinoma, its relevance in the neuro‐urological setting, especially during the initial diagnostic evaluation, is not clear [8]. Chronic bladder inflammation in NLUTD may lead to reactive cytological atypia, limiting the specificity of BWC and increasing the risk of false‐positive results [9].

Current guidelines recommend regular surveillance in patients with high‐risk neuro‐urological disorders, particularly those with long‐term catheterisation [2]. However, evidence regarding the necessity and diagnostic yield of BWC during the initial evaluation of patients with neuro‐urological disorders without known risk factors is lacking.

We hypothesised that BWC does not add relevant diagnostic value beyond macroscopic findings at UC and can be safely omitted in the initial evaluation of patients with neuro‐urological disorders. We therefore evaluated its contribution to surgical decision‐making regarding transurethral bladder surgery (TUBS) and quantified its diagnostic accuracy and costs.

## Patients and Methods

### Patients

In a prospective cross‐sectional study, we investigated a consecutive series of patients with LUTS referred for neuro‐urological evaluation who underwent UC and BWC from 2018 to 2023 at our Department of Neuro‐Urology. Inclusion criteria involved patients aged ≥18 years, undergoing first UC with BWC within 6 months of referral for the neurological condition and no gross haematuria at presentation. The study was approved by the local ethics committee, i.e., Kantonale Ethikkomission (KEK) Zürich, Switzerland (study identification number: KEK 2010‐0207/0). All patients included in the study provided written informed consent according to the Helsinki II declaration.

### Investigations

The initial neuro‐urological evaluation included a 3‐day bladder diary, medical history, neuro‐urological examination, uroflowmetry, post‐void residual measurement, renal ultrasound, UC and BWC (regardless of the macroscopic finding during UC), and video‐urodynamics [1, 2]. UC was performed under local anaesthesia in an outpatient setting with a lidocaine lubricating gel (Instillagel® 2 × 10 mL, Farco‐Pharma GmbH, Köln, Germany) that was instilled into the urethra and exposed for 5–10 min. We used a rigid cystoscope in lithotomy position in women and a flexible cystoscope in a supine position in men, respectively. After bladder inspection, we routinely performed a BWC. If tumour‐suspicious lesions were detected in the UC and/or BWC, patients were scheduled for TUBS. Either the macroscopic lesion was resected, or, in case of exclusive suspicious BWC, random cold biopsies were taken under spinal or general anaesthesia. Histological and cytological findings were reported by board certified pathologists and cytologists. Cytology was reported using conventional multi‐tiered diagnostic categories and according to the Paris System for Reporting Urinary Cytology (TPS) with the following editions [10] starting from 2020.

### Study Measures

Tumour‐suspicious lesions were defined in UC as red patches, papillary or polypoid structures, ulcerations or atypical vessel formations. Cytological findings were categorised as clinically positive and negative. Clinically positive findings comprised atypical urothelial cells, suspect of high‐grade urothelial carcinoma, low‐grade urothelial neoplasia, as well as keratinising squamous metaplasia. All other specimens, including negative for high‐grade urothelial carcinoma, were considered clinically negative. Non‐diagnostic or unsatisfactory specimens underwent repeat cytology before final classification.

The primary outcome of interest was to assess whether UC and/or BWC had any impact on the clinical decision to proceed with TUBS. Additionally, in cases of positive UC or BWC and subsequent TUBS, the secondary outcome of interest was the presence of clinically relevant findings in the histological analysis of bladder biopsies. Relevant histological findings were defined as BCa (urothelial or squamous cell carcinoma [SCC]) or precancerous lesions (keratinising squamous metaplasia) according to the WHO Classification of Urinary and Male Genital Tumours [11].

A detailed description of the methodology of cost calculation, including tariff structure assumptions and sensitivity scenarios, is provided in the Supporting Information.

### Statistical Analysis

For descriptive characteristics, frequencies of categorical data and medians with the first and third quartile (Q1–Q3, interquartile range [IQR]) for variables defined as continuous were listed and calculated. Diagnostic accuracy, i.e., sensitivity and specificity, of BWC (index test) in detecting BCa was calculated taking UC as the reference standard [12] with 95% binominal extract (Clopper–Pearson) CIs. Missing data were rare and reported in the statistical tables. As two patients with relevant UC or BWC findings were lost‐to‐follow‐up, sensitivity analyses for diagnostic accuracy were conducted – first assigning both cases to the negative histology group, then assigning both cases to the positive group. Statistical analyses were performed using SPSS®, version 27 (IBM Corp., Armonk, NY, USA).

## Results

A total of 695 neurological patients were evaluated for study inclusion, whereof 581 patients (84%) could be included (Fig. 1).

**Fig. 1:**
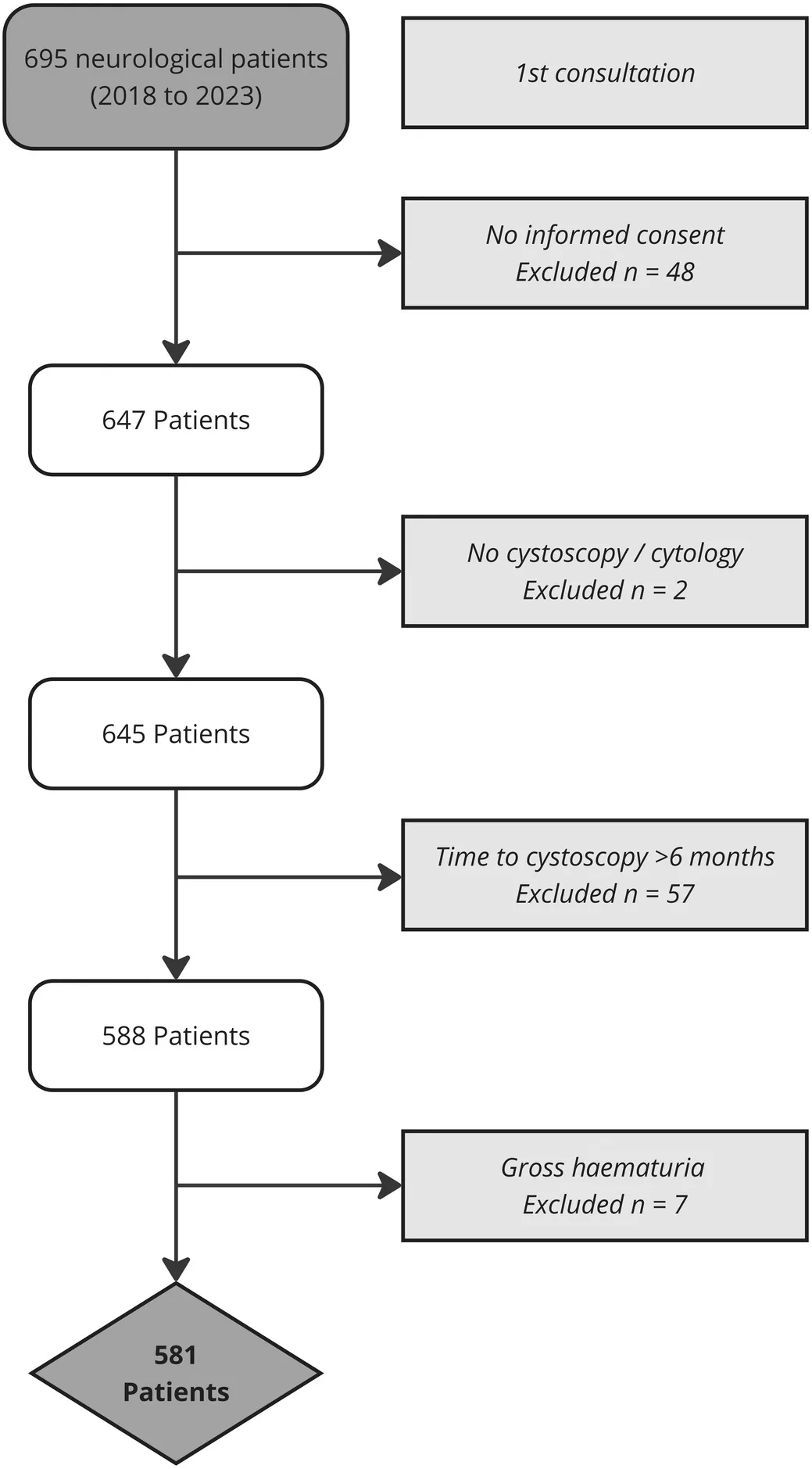
Flow chart of patients included in the analysis from 2018 to 2023 with the exclusion criteria mentioned in a step wise manner.

The median (SD; IQR) age was 62 (16.4; 49–73) years. In all, 274 (47.2%) were women and 307 (52.8%) were men. NLUTD was most frequently caused by SCI, PD, and MS. The patients’ characteristics at presentation are shown in Table 1.

**Table 1:** Patients’ characteristics and bladder management according to neurological disorder.

Neurological disorder	Number of patients, (%)	Age at UC, years, median (IQR)	Sex, *n* (% of overall population)		Bladder management, *n* (% of overall population)		
			Females	Males	Spontaneous voiding	Intermittent self‐catheterisation	Indwelling catheter (suprapubic/transurethral)
Overall	581 (100)	62 (49–73)	274 (47.2)	307 (52.8)	428 (73.7)	54 (9.3)	99 (17.0)
MS	57 (9.8)	54 (44–59)	38 (66.7)	19 (33.3)	44 (77.2)	6 (10.5)	7 (12.3)
PD	90 (15.5)	72 (64–77)	31 (34.4)	59 (65.6)	0	87 (96.7)	3 (3.3)
SCI	156 (26.9)	57 (43–72)	58 (37.2)	98 (62.8)	64 (41.0)	29 (18.6)	63 (40.4)
Complete*	24 (15.4)	51 (34–61)	5 (20.8)	19 (79.2)	3 (12.5)	8 (33.3)	13 (54.2)
Incomplete*	132 (84.6)	60 (44–73)	53 (40.2)	79 (59.8)	61 (46.2)	21 (15.9)	50 (37.9)
Cerebral palsy	9 (1.5)	38 (32–55)	2 (22.2)	7 (77.8)	8 (88.9)	0	1 (11.1)
Spina bifida	10 (1.7)	44 (25–62)	5 (50.0)	5 (50.0)	6 (60.0)	4 (40.0)	0
Cerebrovascular accident	19 (3.3)	73 (68–77)	13 (68.4)	6 (31.6)	13 (68.4)	1 (5.3)	5 (26.4)
Other	240 (41.3)	63 (50–74)	127 (52.9)	113 (47.1)	206 (85.8)	14 (5.8)	20 (8.4)

* Percentage related to total SCI.

In total, 20 (3.4%, 95% CI 2.1–5.3%) patients had relevant UC or BWC findings and 17 (2.9%) underwent TUBS. Out of these, eight patients had a relevant histological result (1.4%, 95% CI 0.6–2.7%) (Table 2, Fig. 2). Six (1.0%) had urothelial carcinoma (three patients with pathological T stage [pT]a low grade and three with pTa high grade). All eight patients with positive histological findings were identified by UC, four of the eight had the initial evaluation less than a year after neurological diagnosis. Two of the eight patients were current smokers at the time of evaluation. The remaining 561 patients (96.6%, 95% CI 94.7–97.9%) had negative UC and BWC results and did not undergo surgery. All 17 (2.9%) patients with tumour‐suspicious lesions in UC had negative cytological findings (Fig. 2, patient details in Table S1). One patient denied surgery despite indication for TUBS and was lost‐to‐follow‐up due to moving abroad. Conversely, three (0.5%) patients had non‐suspicious findings in UC combined with a positive cytology finding (Table 3, Fig. 2). One patient underwent random cold biopsies with a negative histological result. One patient denied surgery and had further follow‐up by BWC and UC, which were negative over a follow‐up period of 4.5 years, and one had further outpatient care in another hospital without repetition of BWC or UC.

**Table 2:** Overview of histological findings after TUBS in patients with NLUTD.

Variable	*N* (%)
TUBS	17 (2.9)
Normal	2
Inflammatory lesions	5
Keratinising squamous metaplasia	2
Urothelial carcinoma	6
pTa low grade	3
pTa high grade	3
Papilloma	1
PUNLMP	1

PUNLMP, papillary urothelial neoplasia of low malignant potential.

**Fig. 2:**
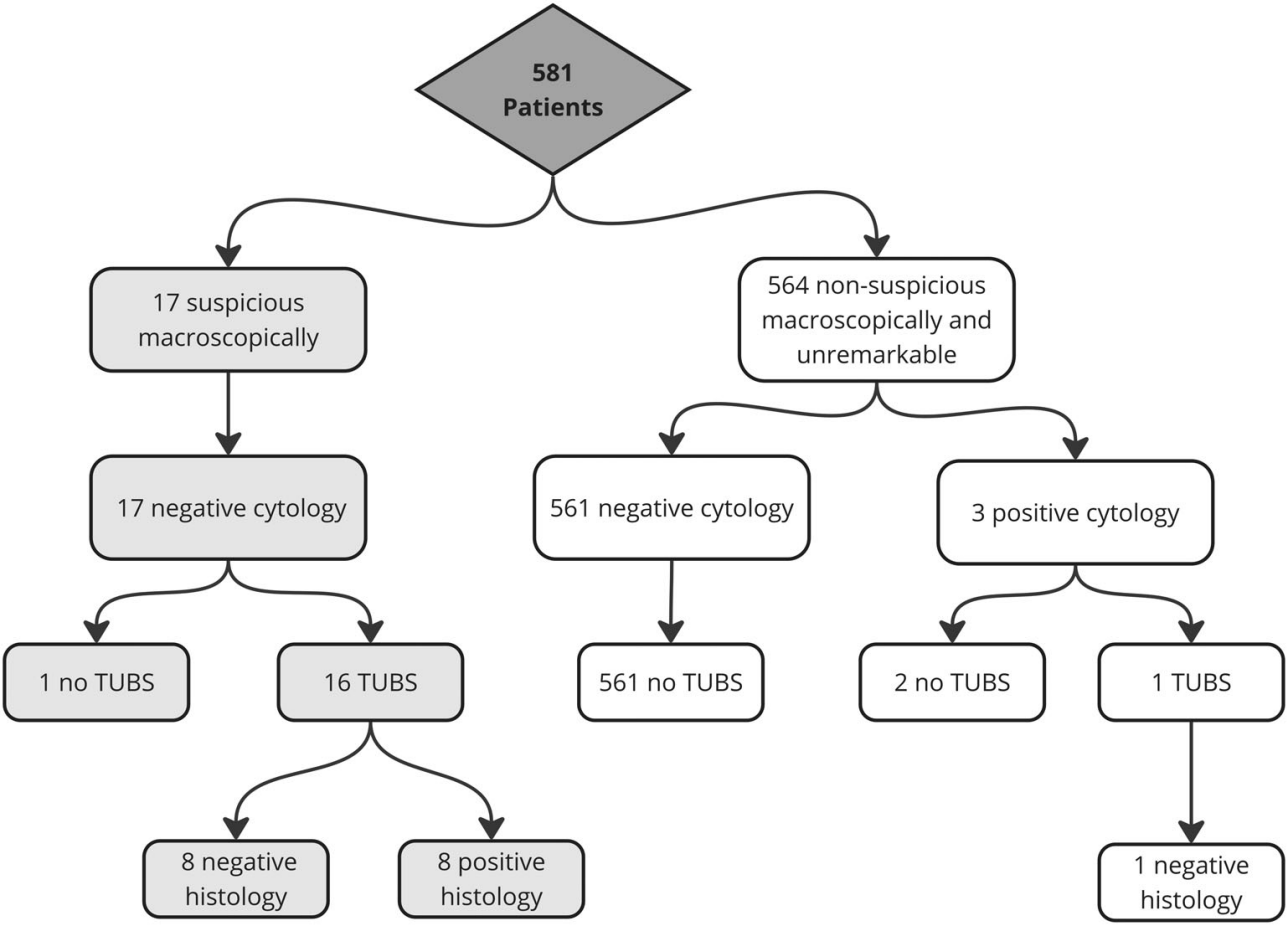
Diagnostic outcomes after evaluation with UC, BWC, and TBS.

**Table 3:** Details of patients with no macroscopic suspicious lesion in UC but positive BWC.

Patient	Sex	Age, years	Neurological disease	Years of diagnosis of neurological disease	Bladder management	Cytological findings	Histological finding after TUBS	Follow‐up
1	Male	74	Poliomyelitis	65	Spontaneous voiding	Papillary urothelial neoplasia	Refused surgery	No surgical or cytological follow‐up
2	Male	63	Tetraspasticity after encephalitis	61	Spontaneous voiding	Atypical urothelial cells with high suspicion of high‐grade urothelial cancer cells	Negative (inflammation)	Regular, CT scan negative for suspicious lesions in the upper urinary tract
3	Male	54	Complete paraplegia below thoracic 12	33	Intermittent self‐catheterisation	Keratinising squamous metaplasia	Refused surgery	Cytological follow‐up without metaplasia

In complete cases analysis, sensitivity of BWC was 0% (95% CI 0.0–36.9%) and specificity was 99.7% (95% CI 98.7–>99.9%). Two sensitivity scenarios were applied, to handle two cases with tumour‐suspicious findings (one in UC and one in BWC) that were lost‐to‐follow‐up. In both scenarios, the third patient with 4.5 years of follow‐up with negative UC and BWC was considered a false positive. Sensitivity in scenario one (both cases negative) and two (both cases positive) was 0% (95% CI 0.0–36.9%) and 10.0% (95% CI 0.3–44.5%), respectively, whereas specificity was 98.5% (95% CI 98.5–99.9%) and 99.7% (95% CI 98.7–>99.9%) (Table S2).

Detailed cost analyses across different prevalence and sensitivity scenarios are presented in the Fig. S1. Adding BWC to UC increased the screening costs by 22.8% under the current Swiss tariff structure.

## Discussion

In the initial evaluation of our patients with neuro‐urological disorders, all positive histological findings were identified by UC. Conversely, positive BWC without tumour‐suspicious lesions in UC did not lead to the detection of relevant histological findings or change of clinical management. Thus, our study clearly indicates that routine use of BWC in addition to UC is not justified and can be omitted in the initial evaluation of our patients with neuro‐urological disorders without compromising safety.

In the general population, UC is the primary diagnostic tool recommended by the European Association of Urology (EAU) guidelines for the detection of BC, with BWC serving as a complementary, non‐invasive diagnostic method [12]. BWC has a reported sensitivity of up to 84% for high‐grade urothelial carcinoma, whereas its sensitivity for low‐grade tumours is significantly lower (16%) [6]. Particularly for carcinoma *in situ* (CIS), BWC achieves sensitivities ranging from 28% to 100% [13]. Despite these promising numbers, the population with NLUTD warrants consideration with a distinct clinical context.

Patients with NLUTD, especially those with SCI, might have an upon 6.7‐fold increased risk of developing BCa compared to the general population, with an estimated risk of SCC of up to 36.8% in patients with SCI [14, 15, 16]. Chronic bladder irritation due to indwelling catheters, recurrent UTIs, and bladder stones are considered contributing risk factors for the altered oncological profile [4, 17]. The tumour spectrum in this population tends to be dominated by SCC, which is typically diagnosed at a more advanced stage and is associated with a worse prognosis and higher mortality rate [18, 19]. A follow‐up by UC in these patients is therefore recommended, but the diagnostic yield of the BWC remains controversial [4].

In our cohort undergoing the initial diagnostic evaluation, we identified BCa only in patients with suspicious macroscopic abnormalities. No proven BCa was found in patients with only relevant cytological findings, although it has to be mentioned that two patients refused TBS. Nevertheless, these results suggest that in the context of an initial diagnostic evaluation, BWC alone will generally not lead to relevant clinical consequences in the absence of macroscopic abnormalities. Also, our cost analysis shows that BWC added to UC increases screening costs by 22.8% under the current Swiss tariff structure without improving case detection. Even under conservative sensitivity assumptions, the additional cost to detect one extra case remains substantial. As prevalence decreases, the cost per detected case increases considerably, further calling the economic justification for routine BWC in the initial evaluation into question.

Our findings are in line with previous studies in the general population, which demonstrated that treatment decisions in urothelial carcinoma are primarily guided by UC findings, with BWC serving as an adjunctive rather than decisive diagnostic tool [20, 21]. We demonstrated high specificity but low sensitivity for histological verification of BC, which is consistent with the general population's evidence for low‐grade urothelial carcinoma. Given the low sensitivity with CIS <50%, the lack of true positives, and the robustness of the sensitivity of results to lost‐of‐follow‐up handling, our findings support omitting routine BWC from the initial diagnostic evaluation of patients with neuro‐urological disorders without gross haematuria.

Although BWC may play a role in the detection of SCC, its sensitivity in this context remains unclear. Cytological identification of SCC relies on the detection of large, pleomorphic squamous cells with keratinised cytoplasm or hyperchromatic nuclei. However, tumour grade, specimen quality, and cytologist experience significantly influence diagnostic accuracy. Therefore, the detection of low‐grade or early‐stage SCC by BWC remains limited [10]. Matsumoto et al. [22] emphasised the high rate of false‐positive cytological findings in the context of chronic bladder irritation, further reducing the specificity of BWC in patients with NLUTD.

In our cohort, no malignant tumours were detected solely based on cytology. Moreover, half of the macroscopically suspicious lesions did not show any malignant histological finding. Therefore, we strongly recommend that clinical decision‐making during the initial evaluation of patients with neuro‐urological disorders rely on UC findings only.

Importantly, our findings cannot be generalised to follow‐up and high‐risk settings. The EAU Neuro‐Urology Guidelines recommend regular UC monitoring in patients with NLUTD and long‐term catheterisation due to the increased risk of bladder malignancy [2]. In this context, BWC may still be considered as a complementary tool, particularly when cystoscopic visibility is impaired by chronic inflammation, bladder wall deterioration, or the presence of bladder stones. Ideally, UC should be repeated after optimisation of conditions to allow for an accurate assessment of the macroscopic bladder status.

Moreover, the optimal follow‐up interval and screening strategy in patients with neuro‐urological disorders remain a matter of ongoing debate [23]. Current guidelines lack a clear consensus, and risk stratification models based on individual patient characteristics, including catheterisation type and duration, are still under development. Notably, routine screening for urothelial cancer in the general population is not recommended [12], highlighting the need for a balanced, individualised approach in patients with neuro‐urological disorders as well.

This study was based on a sample that is likely to be representative, with data available from 93% of the eligible population that went through the clinic during our study period. There is a potential interobserver bias in both UC and BWC analysis. However, in this study we believe that the impact of observers should be minor due to harmonisation of internal clinic and laboratory procedures. Inflammatory changes secondary to chronic catheterisation or recurrent UTIs may further complicate cytological interpretation, leading to false‐positive or inconclusive results. To minimise the impact of these factors, we excluded patients who received the UC >6 months after referral, but applicability of the results to the excluded group is unclear. Further, not all patients underwent BWC at a standardised interval following diagnosis of their neurological condition, so the study was not powered to investigate time since diagnosis in detail. Moreover, the three patients with positive BWC or suspicious UC, respectively, and missing histological proof leave some uncertainty regarding diagnostic accuracy, but sensitivity analysis confirmed that the results were robust to the handling of these cases.

Future research should aim to refine the diagnostic algorithm for patients with neuro‐urological disorders at risk of BC. Advanced imaging techniques and novel urine biomarkers may eventually surpass the diagnostic accuracy of BWC and improve risk stratification. Until then, selective use of BWC in high‐risk patients, such as those with long‐term indwelling catheters and chronic inflammation, appears reasonable. At least, the results outlined support an adaptation of the current neuro‐urology guidelines recommending that BWC may be omitted from the initial diagnostic evaluation of patients with neuro‐urological disorders.

## Conclusions

Our data indicates that in the initial evaluation of patients with neuro‐urological disorders without gross haematuria, BWC is not warranted, and omission would save relevant costs. Macroscopic UC abnormalities remain the primary driver of clinical decision‐making. However, in follow‐up and high‐risk settings, BWC may retain a complementary role in surveillance and early detection of bladder malignancies warranting appropriately designed prospective studies.

## Funding

None.

## Disclosures of Interest

None.

## Author Contributions

Conception and design: Lehner, Kessler. Data analysis and interpretation: Lehner, Dugas, Anderson, Kessler. Data acquisition: Lehner, Dugas. Drafting manuscript: Lehner. Critical revision of the manuscript for scientific and factual content: All. Supervision: Kessler.

## Supporting information


**Fig. S1.** Screening cost per case detected by UC only (solid lines) and UC with BWC (dashed lines), under different scenarios regarding prevalence of urothelial pathology and sensitivity. The prevalence scale on the *x*‐axis is inverted to display the most to least favourable cost scenarios. The dotted vertical line reflects the prevalence of relevant urothelial pathology observed in the present population, 1.4%. The graph on the left displays the full *y*‐axis range to have an overview of the costs even in the least‐favourable scenarios. The graph on the right has the *y*‐axis truncated for clearer visualisation of the more favourable cost scenarios.
**Table S1.** Details of patients with suspicious macroscopic lesions in UC.
**Table S2.** Bladder washing cytology results cross‐tabulated by UC and bladder histology results (reference standard). All cases with macroscopic lesions (positive UC) and positive BWC findings are referred to surgical follow‐up with histological confirmation. As three cases with suspicious findings (one in UC, two in BWC) did not undergo TUBS, and therefore did not have histological confirmation, two scenarios are presented regarding test sensitivity and specificity: Scenario 1 – both cases lost‐to‐follow‐up belong to ‘negative histology’ group. Scenario 2 – both cases lost to follow‐up belong to the ‘positive histology’ group. The third patient who refused TUBS but had negative UC and BWC follow‐up over 4.5 years was considered a false positive and put in the group ‘Positive BWC & Negative UC or Histology’ for both scenarios.

## Data Availability

The data that support the findings of this study are available from the corresponding author upon reasonable request.
