# Supplementary material for: Bladder washing cytology in the initial evaluation of patients with neuro‐urological disorders: to do or not to do?
Source: BJU Int. 2026 Aug 16;138(5):860–6. doi: 10.1111/bju.70422 (PMC13655198; doi:10.1111/bju.70422)
Supplement: Supplementary file 1 [file BJU-138-860-s001.docx]

***Supplementary table 1.* Details of patients with suspicious macroscopic lesions in urethro-cystoscopy.**

| **Patient** | **Sex** | **Age** | **Neurological Disease** | **Years of diagnosis of neurological disease** | **Bladder management** | **Histological finding after TUBS** | **Follow-up** |
| --- | --- | --- | --- | --- | --- | --- | --- |
| **1** | Female | 75 | Myelomalzia, bleeding Th5-S2 | 0 | Spontaneous voiding | pTa low-grade | According to EAU guidelines |
| **2** | Female | 59 | Lumbar spinal stenosis | 0 | Spontaneous voiding | Keratinizing squamous metaplasia | Cystectomy due to precancerous state and refractory storage symptoms |
| **3** | Male | 72 | Claudicatio spinalis, polyneuropathy | Not available | Spontaneous voiding | Inflammatory lesions | - |
| **4** | Female | 68 | Contusio spinalis | 0 | Transurethral catheter | Papilloma | - |
| **5** | Female | 85 | Poliomyelitis, crowded dens syndrome | 80 | Spontaneous voiding | pTa low-grade | According to EAU guidelines |
| **6** | Male | 67 | Spinal canal stenosis | 2 | Spontaneous voiding | PUNLMP | - |
| **7** | Female | 66 | Disc herniation Th11/12, L4/5 | 13 | Self-catheterization | Inflammatory lesions | - |
| **8** | Male | 42 | Disc herniation L5/S1 | 0 | Spontaneous voiding | Inflammatory lesions | - |
| **9** | Male | 80 | Parkinson’s disease | 5 | Spontaneous voiding | Follicular cystitis | - |
| **10** | Male | 72 | Parkinson’s disease | 31 | Spontaneous voiding | pTa low-grade | According to EAU guidelines |
| **11** | Male | 61 | Parkinson’s disease | 17 | Spontaneous voiding | Normal | - |
| **12** | Male | 59 | Incomplete paraplegia below Th11 | 0 | Spontaneous voiding | pTa high-grade | According to EAU guidelines |
| **13** | Male | 82 | Spinal canal stenosis L3/4 | 0 | Spontaneous voiding | pTa high-grade | According to EAU guidelines |
| **14** | Male | 60 | Parkinson’s disease | 7 | Spontaneous voiding | pTa high-grade | According to EAU guidelines |
| **15** | Male | 29 | Complete tetraplegia below C5 | 12 | Spontaneous voiding | Refused surgery | Lost-to-follow-up, moved abroad |
| **16** | Female | 51 | Multiple sclerosis | 1 | Spontaneous voiding | Inflammatory lesions | - |
| **17** | Male | 73 | Incomplete paraplegia below Th6 | 25 | Self-catheterization | Keratinizing squamous metaplasia | Regular cystoscopic follow-up |

*Abbreviations: C = cervical; EAU = European Association of Urology; L = lumbar; Th = thoracic; TUBS = transurethral bladder surgery; PUNLMP = papillary urothelial neoplasm of low malignant potential.*

***Supplementary table 2.* Bladder washing cytology (BWC) results cross-tabulated by urethro-cystoscopy (UC) and bladder histology results (reference standard).** All cases with macroscopic lesions (positive UC) and positive BWC findings are referred to surgical follow-up with histological confirmation. As 3 cases with suspicious findings (1 in UC, 2 in BWC) did not undergo transurethral bladder surgery (TUBS), and therefore did not have histological confirmation, two scenarios are presented regarding test sensitivity and specificity: Scenario 1 – both cases lost-to-follow-up belong to ‘negative histology’ group. Scenario 2 – both cases lost to follow-up belong to the ‘positive histology’ group. The third patient who refused TUBS, but had negative UC and BWC follow-up over 4.5 years was considered a false positive and put in the group ‘Positive BWC & Negative UC or Histology’ for both scenarios.

| **No Scenario** | *Positive UC & histology* | *Negative UC or histology* |
| --- | --- | --- |
| *Positive BWC*  *Negative BWC* | 0  8 | 1  569 |
| **Scenario 1** | *Positive UC & histology* | *Negative UC or histology* |
| *Positive BWC*  *Negative BWC* | 0  8 | 3*  570 |
| **Scenario 2** | *Positive UC & histology* | *Negative UC or histology* |
| *Positive BWC*  *Negative BWC* | 1  9 | 2*  569 |

* One patient who refused TUBS with negative UC and BWC follow-up over 4.5 years was considered a false positive.

**No Scenario:**

Sensitivity 🡪 0.0% (95% CI: 0.0-36.9%)

Specificity 🡪 99.8% (95% CI: 99.0->99.9%)

**Scenario 1:**

Sensitivity 🡪 0.0% (95% CI: 0.0-36.9%)

Specificity 🡪 99.5% (95% CI: 98.5->99.9%)

**Scenario 2:**

Sensitivity 🡪 10.0% (95% CI: 0.3-44.5%)

Specificity 🡪 99.7% (95% CI: 98.7->99.9%)

**Bladder Washing Cytology – Cost Scenarios**

**Methods**

Screening costs of bladder washing cytology per case detected were calculated based on numbers from the TARDOC (Tarif für ambulante ärztliche Leistungen und Dokumentation) database. The TARDOC database underlies the outpatient physician reimbursement system and defines standardized tariff structure used to bill medical services in Switzerland. Costs were first calculated using the exact numbers from the study population, and then over various scenarios for the prevalence of relevant urothelial pathology in the population undergoing an initial neuro-urological work-up. At the time of TARDOC database access in early February 2026, using the conversion of one TARDOC point=0.91 CHF, and an exchange rate of 1 CHF=1.075 Euros, a urethro-cystoscopy (UC) costs CHF 530 or €570 and a bladder washing cytology (BWC) costs CHF 121 or €130. Costs of follow-up assessments for positive diagnostic findings (TURB, biopsies, histology) were not included in the present analysis as the substantial variation in surgical costs that is dependent on patient characteristics such as comorbidities limits the scope for generalizability. Prevalence scenarios were chosen based on estimates from the current population, assuming the case mix of patients admitted to the clinic for a first neuro-urological work-up, as well as bladder cancer rates will remain relatively stable in Switzerland in the coming years (1). The three diagnostic sensitivity scenarios, 0.99, 0.9, and 0.5 were selected to be roughly in line with UC performance and lower 95% confidence intervals (CI) under the most conservative loss-to-follow-up scenario: sensitivity 0.90 (95% CI: 0.55->0.99).

**Results**

In the current population under 2026 Swiss medical tariff structure, including the BWC as an adjunct to the UC increased the screening costs by 22.8%. Absolute screening costs per true positive identified would have been €41'412 for UC alone and €50'872 under the combined UC and BWC protocol (n=8 true positives, 581 cases screened). Under the sensitivity scenario where both cases lost-to-follow-up were true positives, the cost per true positive detected using UC alone (n=9) would have been €36'810, while the cost per true positive detected using UC and BWC (n=10) would have been €40'697, reflecting a 10.6% increase in costs with the addition of BWC to detect one additional case.

*Supplementary Figure 1* examines scenarios regarding screening cost per case detected over different scenarios for prevalence of urothelial pathology. Although the relative relationship between cost per case detected remains stable when the two test strategies are assumed to have the same sensitivity, with a 22.8% increase in expenses with the addition of BWC as an adjunct, the costs for each new case detected start to markedly increase as the expected prevalence of relevant pathology decreases. While there is only a 11% increase in the cost for each new case detected when the prevalence of relevant pathology decreases from 5% to 4.5%, there is a 50% increase in cost when the prevalence of urothelial pathology decreases from 1.5 to 1% and a 100% increase in cost when it drops from 1% to 0.5%.

***Supplementary Figure 1*.** Screening cost per case detected of urethro-cystoscopy (UC) only (solid lines) and UC with bladder washing cytology (BWC) (dashed lines), under different scenarios regarding prevalence of urothelial pathology and sensitivity. The prevalence scale on the x-axis is inverted to display the most to least favorable cost scenarios. The dotted vertical line reflects the prevalence of relevant urothelial pathology observed in the present population, 1.4%. The graph on the left displays the full y-axis range to have an overview of the costs even in the least-favorable scenarios. The graph on the right has the y-axis truncated for clearer visualization of the more favorable cost scenarios.

**Limitation**: This analysis does not cover the costs of follow-up assessments that were triggered by positive diagnostic findings, so absolute costs to reach a 'true positive' diagnosis are underestimated for both the UC only and the UC with BWC scenarios. The relationship in relative costs may shift in favor of BWC if the costs of follow-up investigations are accounted for, as BWC results in fewer referrals for follow-up investigations requiring surgery and histological testing.

Reference:

1.	Trächsel B, Rapiti E, Feller A, Rousson V, Locatelli I, Bulliard JL. Predicting the burden of cancer in Switzerland up to 2025. PLOS Glob Public Health. 2022;2(10):e0001112.
